# Pathological discrimination between luteinized thecoma associated with sclerosing peritonitis and thecoma

**DOI:** 10.1097/MD.0000000000033911

**Published:** 2023-06-09

**Authors:** Jia Liu, Jia Wei, Yiqun Yang, Juncheng Wei

**Affiliations:** a Department of Obstetrics & Gynecology, Tongji Hospital, Tongji Medical College, Huazhong University of Science & Technology, Hubei Province, China.

**Keywords:** ovarian neoplasms, pathology, sex cord-gonadal stromal tumors

## Abstract

Similarities between luteinized thecoma associated with sclerosing peritonitis (LTSP) and thecoma, cause difficulty in clinical differential diagnoses. To improve the situation, we selected 10 specified molecular pathological markers that are frequently used in clinical pathology of ovarian sex cord-stromal tumors to determine whether they exert a discriminatory effect.

**Methods:**

Applying immunohistochemistry, we analyzed the expression of alpha-1,6-mannosylglycoprotein 6-beta-n-acetylglucosaminyltransferase B (MGAT5B), nuclear receptor coactivator 3 (NCOA3), proliferation marker protein Ki-67 (MKI67), estrogen receptor, progesterone receptor, Vimentin, receptor tyrosine-protein kinase erbB-2, Catenin beta-1 (β-Catenin), CD99 antigen (CD99) and Wilms tumor protein (WT1) in 102 cases of diseases containing 11 LTSP and 91 thecoma. Whole-exome sequencing and fluorescence in situ hybridization were used to examine the MGAT5B-NCOA3 fusion gene in LTSP. Statistical analysis was performed using *t* test, one-way analysis of variance test, and post hoc test.

**Results:**

Six significant markers were verified for the discrimination between LTSP and thecoma, containing 4 upregulating indicators MGAT5B, NCOA3, MKI67, β-Catenin, and 2 downregulating markers CD99 and WT1 in luteinized cells. In addition, the MGAT5B-NCOA3 fusion gene was identified in LTSP for the first time with significantly rich expression compared to thecoma.

**Conclusions:**

We verified 6 significant molecular pathological markers containing MGAT5B, NCOA3, MKI67, β-Catenin, CD99, and WT1 and identified MGAT5B-NCOA3 fusion gene in LTSP; this work will help clinicians to discriminate between medical conditions and treat patients accurately.

## 1. Introduction

Thecoma is a very uncommon disease representing 1~2% of all ovarian tumors.^[1,2]^ A rarer variant of ovarian neoplasm is luteinized thecoma associated with sclerosing peritonitis (LTSP).^[3,4]^ LTSP belongs to ovarian sex cord-stromal tumors (SCST); it generally arises in young and midlife women (median age: 28 years), and clinically presents as a bilaterally round mass in the abdomen.^[5]^ Unlike the most benign behaviors of thecoma, LTSP manifests as sclerosing peritonitis leading to abdominal distension, ascites, and bowel obstruction. Patients with the aforesaid complications have a poor prognosis.^[4,6–8]^ Microscopically, tumors with constituent uniform cells containing pale-gray cytoplasm and indistinct cell membranes are diagnosed as thecoma.^[9]^ Otherwise, LTSP cells are typical fusiform cells histologically with interspersed clusters of luteinized cells, marked lipoedema, and entrapped ovarian follicles, which concurrently show as single cells in a cellular fibroblastic background.^[10,11]^ Furthermore, LTSP is hypercellular but smaller than thecoma cells with striking mitotic activity.^[3]^ Currently, the differential diagnosis of the 2 subtypes of ovarian SCST needs to be improved as the existing evidence is mostly based on single cases and there is insufficient guidelines regarding the clinicopathology and management of LTSP. Surgery represents the cornerstone of treatment, including hysterectomy and adhesiotomy. Abdominal symptoms are mitigated using hormonal therapy with leuprorelin and tomaxifen.^[6,7,12]^

In this study, we presented the histopathological and molecular features of 102 cases, including LTSP and thecoma. A detailed immunohistochemical analysis was undertaken to explore the pathological distinction between the 2 particular subtypes of ovarian pure stromal tumors. Moreover, we explored this first discovery of the alpha-1,6-mannosylglycoprotein 6-beta-n-acetylglucosaminyltransferase B (MGAT5B) – nuclear receptor coactivator 3 (NCOA3) fusion gene to verify its expression and substantial effect, with the aim of treating patients more effectively.

## 2. Material and methods

### 2.1. Subjects and samples

The study was performed under Tongji Hospital Institutional Review Board approval with permit number TJ-IRB20220925. One hundred and two cases were retrieved from the archives of 4 institutions, including Central Hospital of Wuhan (Wuhan, China), Maternity and Childcare Hospital of Hubei Province (Wuhan, China), Central Hospital of Xiangyang (Xiangyang, China) and Tongji Medical College of Huazhong University of Science & Technology (Wuhan, China), between 2001 and 2020. Patients with LTSP or thecoma were included in this research, regardless of age, concomitant diseases, and treatment approaches. LTSP and thecoma comprised 11 and 91 cases respectively. The unstained tissue sections were 3 to 10 μm thick, formalin-fixed, and paraffin-embedded beforehand by each aforementioned institution.

### 2.2. Immunohistochemistry (IHC)

The tissue sections were stained for MGAT5B (1:200; catalog number 16993; Proteintech, Wuhan, China), NCOA3 (1:50, catalog number 20032, Proteintech), Vimentin (1:200, catalog number 10366, Proteintech), Catenin beta-1 (β-Catenin; 1:200, catalog number 17565, Proteintech), receptor tyrosine-protein kinase erbB-2 (HER2; 1:600, catalog number 18299, Proteintech), proliferation marker protein Ki-67 (MKI67; 1:5000, catalog number 27309, Proteintech), estrogen receptor (ESR; 1:200, catalog number 21244, Proteintech), progesterone receptor (PGR; 1:200, catalog number 25871, Proteintech), CD99 antigen (CD99; 1:200; catalog number abs136282; Absin, Shanghai, China), Wilms tumor protein (WT1; 1:200, catalog number abs135851, Absin), and hematoxylin-eosin as follows. Sections were deparaffinized using xylene and rehydrated in a graded ethanol series. After 15 minutes of pH 6.0 boiled citrate antigen retrieval, sections were incubated using General SP Kit (Mouse/Rabbit Streptavidin-Biotin Detection Systems; catalog number SP9001; ZSGB-BIO, Beijing, China) following the manufacturer’s protocol. Pretitrated dilutions of primary antibodies were incubated overnight at +4°C in 5% skim milk (1:20; catalog number G5002; Servicebio, Wuhan, China), followed by chromogenic detection with a DAB Detection Kit (1:100, catalog number G1212, Servicebio). Staining was imaged using an upright fluorescence microscope (BX53; Olympus, Tokyo, Japan) and a slide scanner (KF-FL-400; KFBIO, Zhejiang, China) with the software KFCloud Viewer (KF-FL-400; KFBIO, Zhejiang, China). Five high-power fields were selected randomly for each case. For comparison, the staining intensity was represented as average optical density (AOD), calculated by integrated optical density, divided by area, which is limited to threshold using the software Image Processing and Analysis in Java 1.8.0 (ImageJ; National Institute of Mental Health, MD).

### 2.3. Whole-exome sequencing

One LTSP case was subjected to whole-exome sequencing using validated protocols at the BGI group (Shenzhen, China). Sequencing data were analyzed and mutations identified using validated bioinformatics methods such as differential expression gene analysis.

### 2.4. MGAT5B-NCOA3 fluorescence in situ hybridization (FISH)

Twelve cases including 7 cases of LTSP and 5 cases of thecoma underwent FISH to identify the MGAT5B-NCOA3 fusion gene using a custom fusion probe (catalog number FP337; HealthCare Biotechnology, Wuhan, China) following the manufacturer’s instructions. Probes for detection of the MGAT5B-NCOA3 fusion gene consisted of a dual-color probe mix, composed of bacterial artificial chromosomes mapping to NCOA3 (orange) and MGAT5B (green). Briefly, sections were deparaffinized at 68°C for 15 minutes, hyalinized at 90°C for 20 minutes, and digested at 37°C with a prepared protease working solution. Subsequent hybridization took place using an In Situ Hybridization Instrument (SH2000; RUICHENG, Zhejiang, China) with the following procedure: denaturing at 85°C for 5 minutes followed by hybridization at 42°C for 16 hours. Following the incubation period, the slides were rinsed and stained with 4’,6-diamidino-2-phenylindole (catalog number CL004, HealthCare Biotechnology), mounted and examined under an upright fluorescence microscope (BX53, Olympus). The positive incidence was calculated as the number of positive cells exhibiting a yellow fluorescent signal divided by the total number of cells in 1 high-power field.

### 2.5. Statistical analysis

IBM Statistical Product and Service Solutions 26.0 (SPSS Inc., Chicago, IL) and GraphPad Prism 8.0 software (GraphPad Software Inc., San Diego, CA) were used for statistical analysis. The quantitative data were represented by mean ± SD and were analyzed using *t* test, one-way analysis of variance test, and post hoc test. *P* < .05 was considered statistically significant.

## 3. Results

### 3.1. Immunohistochemical findings

**Figure 1.**
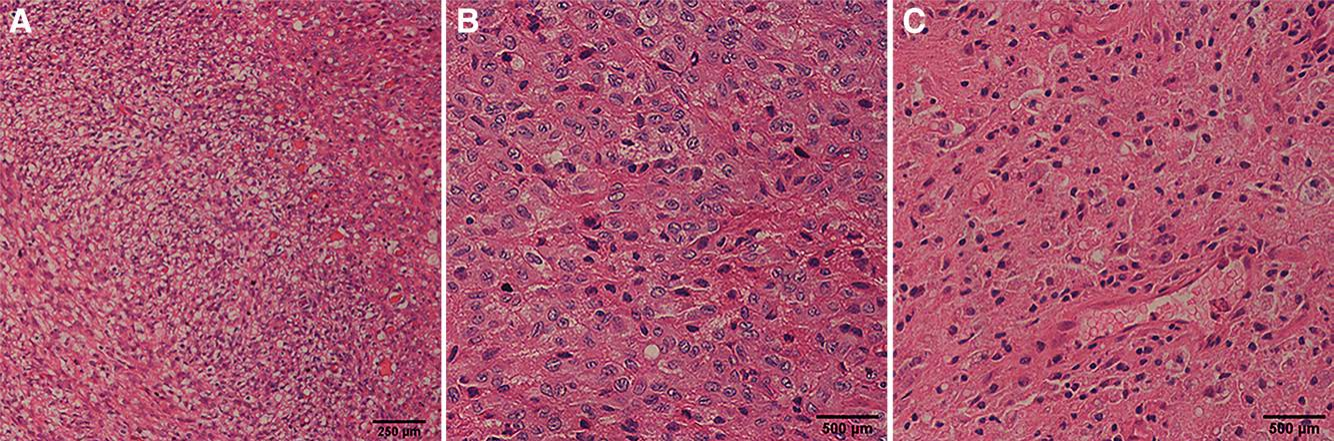
Cells of luteinized thecoma associated with sclerosing peritonitis assessed by hematoxylin-eosin staining. (A) Luteinized cells are present throughout the background but apparent in the edematous area; obvious transition is between the spindle cellular area and edema area. Scale bar, 250 μm. (B) Small clusters of luteinized cells with rounded nuclei and eosinophilic to scant cytoplasm among the spindle cells. (C) dense spindle cells. Scale bar, 500 μm.

Interspersed among the spindle cells were variable numbers of rounded or elliptic cells that were present individually, in small formed clusters, or occasionally in larger chunks. They were present in all LTSP cases, as the most striking feature. The rounded or elliptic cells had variably obvious clearing of the cytoplasm, showing as pale or eosinophilic cytoplasm, consistent with luteinization. In these luteinized cells, prominent lipids were present and viewed as vacuoles and edema, and their nuclei were round or oval and more hyperchromatic than those of the spindle cells, with one or more evident nucleoli (Fig. 1).

The immunohistochemical results are summarized in Table 1. Ten predetermined molecular pathological markers that are commonly used in clinical diagnosis were detected by IHC in cells divided into the 2 groups of LTSP and thecoma, containing 11 and 91 cases, respectively. Higher AOD values of MGAT5B (*P* < .001), NCOA3 (*P* < .001), MKI67 (*P* < .001), and β-Catenin (*P* < .001) were found in the LTSP group compared to the thecoma group; while there were no statistical differences for ESR (*P* = .76), PGR (*P* = .99), Vimentin (*P* = .66), and HER2 (*P* = .85) between the 2 groups. Furthermore, the AOD values of CD99 (*P* = .013) and WT1 (*P* < .001) were significantly higher in thecoma cells compared to luteinized cells.

**Table 1:** Comparison of molecular pathological markers in luteinized thecoma associated with sclerosing peritonitis and thecoma.

Antibody	AOD*		*P* †
	LTSP (n = 11)	Thecoma (n = 91)	
MGAT5B	1.27 ± 0.65	0.94 ± 0.25	<.001
NCOA3	1.13 ± 0.44	0.87 ± 0.16	<.001
ESR	0.95 ± 0.46	0.97 ± 0.18	.76
PGR	0.99 ± 0.43	0.99 ± 0.21	.99
MKI67	1.27 ± 0.13	1.18 ± 0.08	<.001
Vimentin	1.03 ± 0.18	1.05 ± 0.14	.66
HER2	1.00 ± 0.20	1.01 ± 0.19	.85
β-Catenin	1.07 ± 0.18	0.91 ± 0.17	<.001
CD99	1.00 ± 0.25	1.09 ± 0.14	.013
WT1	0.97 ± 0.13	1.13 ± 0.17	<.001

* AOD data are mean ± SD, calculated by integrated optical density divided by threshold area.

† *P* values presented for group comparison are *t* tests.

AOD = average optical density, CD99 = CD99 antigen, ESR = estrogen receptor, HER2 = receptor tyrosine-protein kinase erbB-2, LTSP = luteinized thecoma associated with sclerosing peritonitis, MGAT5B = alpha-1,6-mannosylglycoprotein 6-beta-n-acetylglucosaminyltransferase B, MKI67 = proliferation marker protein Ki-67, NCOA3 = nuclear receptor coactivator 3, PGR = progesterone receptor, WT1 = Wilms tumor protein, β-Catenin = Catenin beta-1.

**Figure 2.**
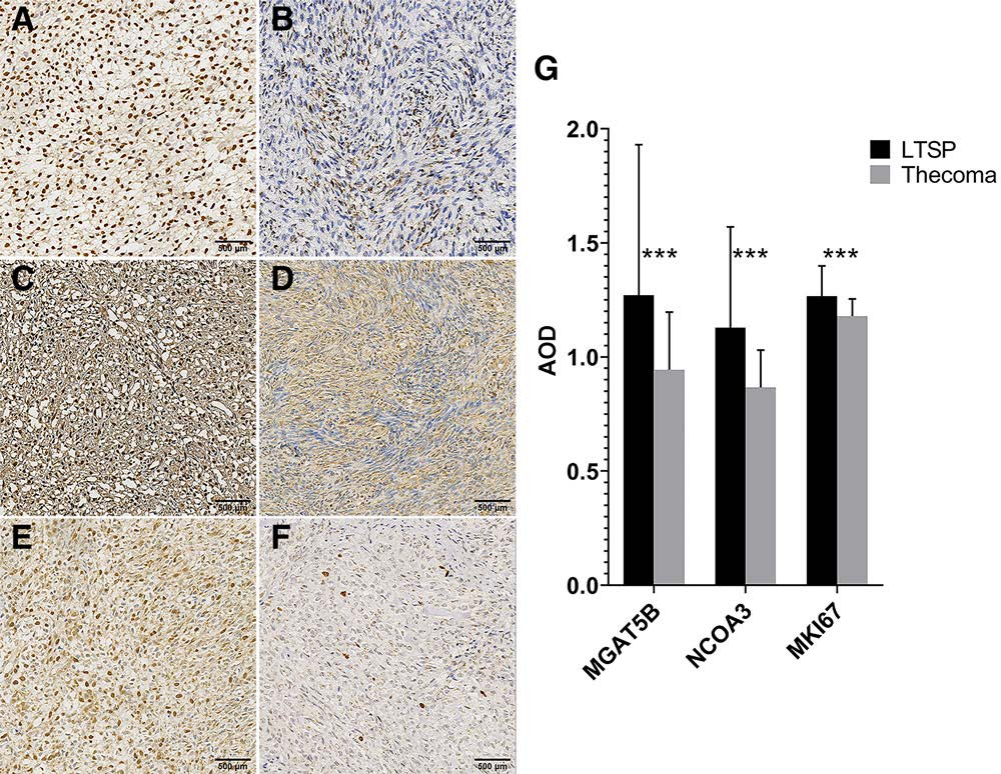
Molecular pathological markers show significant ascendant expression in LTSP. (A–F) Immunohistochemical staining of alpha-1,6-mannosylglycoprotein 6-beta-n-acetylglucosaminyltransferase B (MGAT5B), nuclear receptor coactivator 3 (NCOA3) and proliferation marker protein Ki-67 (MKI67) are respectively (A, C, E) diffusely positive in the luteinized cells but (B, D, F) weak to moderate in the thecoma cells. (G) Statistical analysis of the 3 markers. Data are expressed as mean ± SD. n = 9 to 11 sections per marker (G, LTSP); n = 81 to 85 sections per marker (G, Thecoma). ***P < .001 (*t* test). Scale bar, 500 μm. AOD = average optical density, LTSP = luteinized thecoma associated with sclerosing peritonitis.

**Figure 3.**
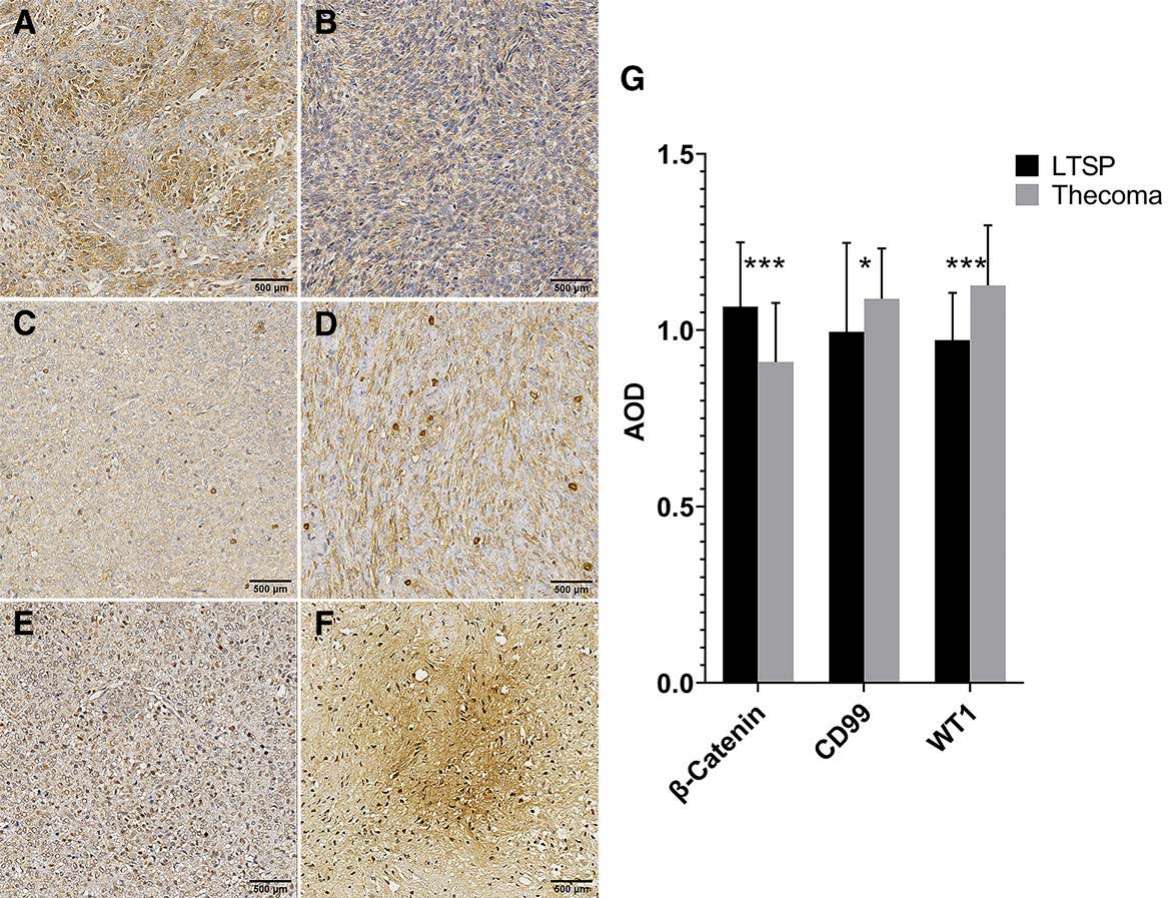
Molecular pathological markers show significant differential expression between LTSP and thecoma. (A and B) Immunohistochemical staining of the luteinized cells is (A) diffusely positive for Catenin beta-1 (β-Catenin) and the thecoma cells are (B) negative with β-Catenin. (C–F) CD99 antigen (CD99) and Wilms tumor protein (WT1) are (C, E) weak in the luteinized cells but (D, F) strongly positive in the thecoma cells. (G) Statistical analysis of the 3 markers. Data are expressed as mean ± SD. n = 9 to 10 sections per marker (G, LTSP); n = 81 to 87 sections per marker (G, Thecoma). *P < .05, ***P < .001 (*t* test). Scale bar, 500 μm. AOD = average optical density, LTSP = luteinized thecoma associated with sclerosing peritonitis.

The luteinized cells were diffusely positive for MGAT5B and NCOA3 with strong staining intensity; meanwhile, their nuclei were focally MKI67 positive (Fig. 2A, C, and E). However, the thecoma cells showed lower staining and fewer chromatic nuclei for the 3 markers (Fig. 2B, D, and F). The expression of MGAT5B, NCOA3, and MKI67 was statistically significant (Fig. 2G). There was focal or diffuse nuclear ESR and PGR immunoreactivity with weak to moderate intensity in both luteinized cells and thecoma cells (see Supplementary Figure S1, Supplemental Digital Content, http://links.lww.com/MD/J40, A–D; which illustrates the expression of ESR and PGR in LTSP and thecoma). For the other 5 molecular pathological markers, the LTSP and thecoma cells showed both weak to moderate cytoplasmic positivity, focally or diffusely, for Vimentin and HER2 (see Supplementary Figure S1, Supplemental Digital Content, http://links.lww.com/MD/J40, E–H; which illustrates the expression of Vimentin and HER2 in LTSP and thecoma). The expression of ESR, PGR, Vimentin, and HER2 was nonsignificant (see Supplementary Figure S1, Supplemental Digital Content, http://links.lww.com/MD/J40, I; which illustrates the statistical results of the 4 markers). The luteinized cells were positive but thecoma cells were negative with β-Catenin (Fig. 3A and B). However, the thecoma cells showed significantly higher staining intensity in the cytoplasm and nuclei respectively for CD99 and WT1 compared to the LTSP group (Fig. 3C–F). The expression of β-Catenin, CD99, and WT1 were statistically significant (Fig. 3G).

### 3.2. Gene feature

**Figure 4.**
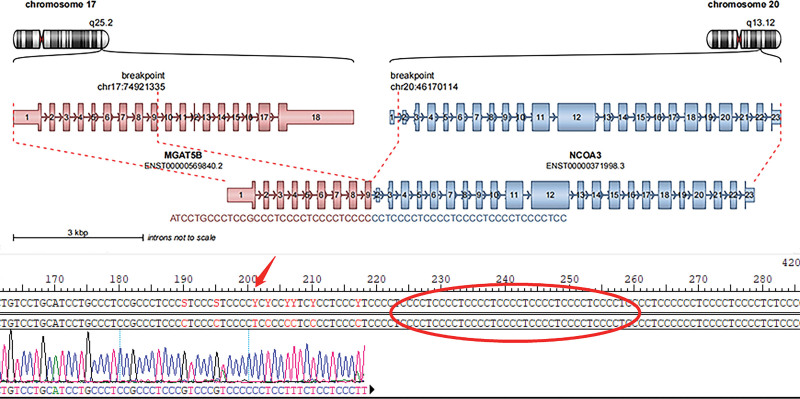
MGAT5B-NCOA3 fusion gene in LTSP of the ovary. Schematic representation of the MGAT5B-NCOA3 fusion transcript including the exons and bases sequence involved. The breakpoint of the 5′ and 3′ partner genes are represented as red dotted lines. LTSP = luteinized thecoma associated with sclerosing peritonitis, MGAT5B = alpha-1,6-mannosylglycoprotein 6-beta-n-acetylglucosaminyltransferase B, NCOA3 = nuclear receptor coactivator 3.

Whole-exome sequencing of one LTSP case revealed a MGAT5B-NCOA3 fusion gene with rich mutation abundance up to 60%, much higher than 0.19% of notch receptor 2 – transmembrane 9 superfamily member 3, which was consequently excluded from subsequent analysis. The fusion gene resulted in a transcript composed of exons 1–9 of MGAT5B (chr17: 74921335) and exons 2–23 of NCOA3 (chr20: 46170114) (Fig. 4). MGAT5B encodes a beta-1,6-N-acetylglucosaminyltransferase that functions in the synthesis of complex cell surface N-glycans, with potential tumorigenesis and metastasis-associated effect.^[13,14]^ Furthermore, MGAT5B may play a part in estrogen-related receptors (ERRs) signaling pathway.^[15]^ NCOA3 encodes a protein with histone acetyltransferase activity, recruiting p300/CBP-associated factor and cAMP-responsive element binding protein as part of a multisubunit coactivation complex.^[16–18]^ In this fusion, the 3′-end of MGAT5B joined to the 5′-UTR of NCOA3 81 Kb before the coding region resulting in an out-frame fusion. Without the production of effective fusion exons, this fusion gene may not code a functional protein (see Supplementary Figure S2, Supplemental Digital Content, http://links.lww.com/MD/J41, which illustrates the fused sequences of MGAT5B and NCOA3 gene fragments).

**Figure 5.**
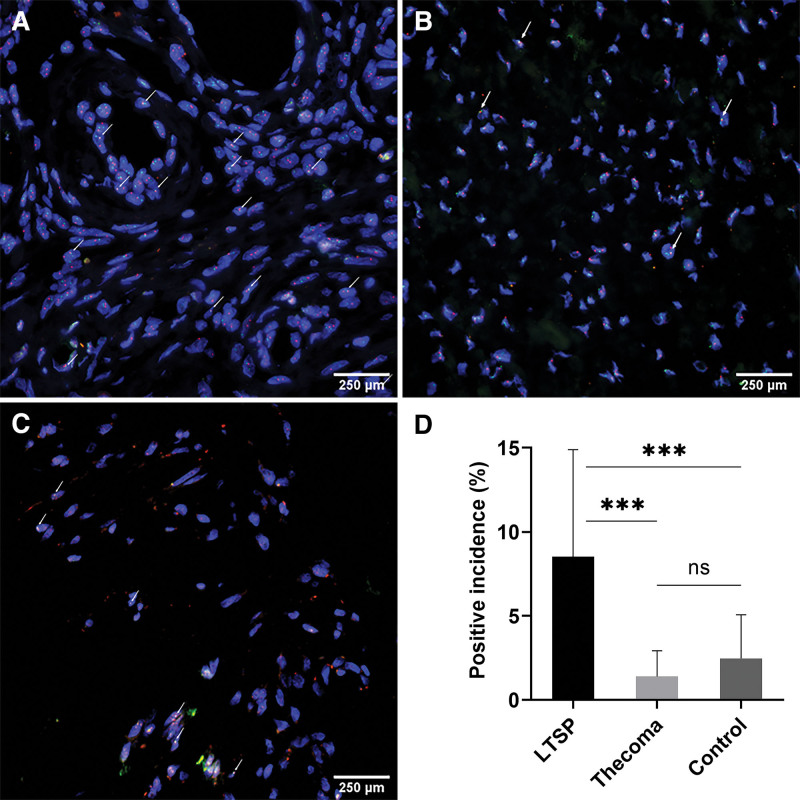
MGAT5B-NCOA3 fusion gene is most common in LTSP. (A–C) MGAT5B-NCOA3 fusion gene is most common in (A) luteinized cells, but (B) rare in thecoma cells and (C) normal fimbriae tubae cells as assessed by FISH. FISH showing fusion signals for MGAT5B-NCOA3 with the overlapping yellow fluorescent signal (white arrows: orange, NCOA3; green, MGAT5B; yellow, fusion area). (D) Statistical analysis of positive incidence. Data are expressed as mean ± SD percentage. n = 7 cases (D, LTSP); n = 5 cases (D, Thecoma); n = 2 cases (D, Control). ***P < .001 (one-way analysis of variance and post hoc test). Scale bar, 250 μm. FISH = fluorescence in situ hybridization, LTSP = luteinized thecoma associated with sclerosing peritonitis, MGAT5B = alpha-1,6-mannosylglycoprotein 6-beta-n-acetylglucosaminyltransferase B, NCOA3 = nuclear receptor coactivator 3, ns = no significance.

Further to detect the MGAT5B-NCOA3 fusion gene, 12 cases including 7 cases of LTSP and 5 cases of thecoma underwent FISH, with normal fimbriae tubae as the simultaneous negative control (Fig. 5). The mean positive incidence was 8.5% in the luteinized cells, significantly higher compared to the 2 other cell types, 1.4% in the thecoma cells (*P* < .001) and 2.5% in the negative control (*P* < .001), respectively. Furthermore, there was no significant difference between thecoma cells and the negative control group (*P* = .54).

## 4. Discussion

To our knowledge, this study is the first to systematically elucidate the differences in molecular pathological features between thecoma and LTSP at the level of molecules and genes. Our study shows that LTSP cells expressed significantly higher MGAT5B, NCOA3, MKI67, and β-Catenin but lower CD99 and WT1 than thecoma cells according to immunohistochemical staining. In addition, a newly identified MGAT5B-NCOA3 fusion gene was discovered for the first time by whole-exome sequencing and further confirmed by FISH.

Similarities between LTSP and thecoma cells cause difficulty in clinical differential diagnoses. However, with specific molecular pathological markers, the situation may be improved. Moreover, the targeting gene of the noncoding RNA pathway, MGAT5B, may also have an effect on promoting tumorigenesis and metastasis by decreasing N-cadherin-associated cell-cell adhesion.^[14,19]^ In addition, overexpression of NCOA3 promotes tumor cell growth and proliferation through various pathways including NCOA3-specificity protein 1-telomerase reverse transcriptase axis in hepatocellular carcinoma.^[20,21]^ Previous studies have demonstrated that MKI67 could help promote metastasis in breast cancer because of its recognized role in cell proliferation activity.^[22]^ Likewise, β-Catenin acts as one transcription factor, also playing a role in tumorigenesis through constituting adhesion junctions and canonical Wnt signaling pathway.^[23–25]^ In thyroid cancer, scientists found that NCOA3 could improve the survival and invasiveness of tumor cells through the modulation of β-Catenin; whereas in hepatocellular carcinoma, knockdown of NCOA3 inhibited the expression of the Wnt signaling-related genes.^[17,20]^ In fibrosarcoma cells, n-acetylglucosaminyltransferase V, the homolog of MGAT5B, sensitized the stimulation of tyrosine phosphorylation of catenins by growth factors and expression of v-src when highly expressed.^[19]^ Consistent with our current findings, the 4 markers related to tumorigenesis and metastasis were significantly upregulated in LTSP compared to thecoma cells. Under the synergistic effect of these highly expressed markers, LTSP cells showed a more aggressive tendency and active proliferation, thus imitating malignant tumors.^[8,11]^

Noteworthy, we identified a new MGAT5B-NCOA3 fusion gene in this study. As mentioned before, studies have pointed out that the 2 genes may be associated with the estrogen pathway.^[15–18]^ Based on the possible mechanism of recruiting cAMP-responsive element binding protein as part of the multisubunit coactivation complex, researchers found that NCOA3 could act on the ERR signaling pathway in different diseases. Indeed, Yi P et al proposed the mode of interaction between NCOA3 and ESRα could be that each of the ligand-bound ESRα monomers independently recruits one NCOA3 protein via the transactivation domain of ESRα and then the 2 NCOA3s, in turn, bind to different regions of one p300 protein through multiple contacts.^[26]^ Since we find the 2 genes are potentially associated with ESRα pathway this adds to the relevance of understanding the role of MGAT5B-NCOA3 fusion gene in the production of ascites and intraperitoneal adhesion. Reports support the view with the finding that nonsteroidal antiestrogen and gonadotropin-releasing hormone agonists could relieve abdominal symptoms by inhibiting related cytokines transforming growth factor beta, tumor necrosis factor-alpha, and nuclear factor kappa B.^[2,6,7,27]^ Therefore, we assume that patients’ abdominal complications could be accurately treated or prevented by detecting the 2 genes in advance. However, the mechanism of MGAT5B on the ESRα pathway is still unclear. Chisamore et al screened 226 genes containing MGAT5B and NCOA3 that were known to be involved in ESRα, cancer, and ERR signaling in breast cancer, but found no significance for the 2 genes.^[15]^

As for CD99 and WT1, they were significantly higher in thecoma than LTSP cells. The 2 markers are highly expressed in the ovary with both tumor-promoting and oncosuppressor effects. CD99 is reported to be related to immunomodulation, homeostasis, and antitumorigenesis via inhibiting cell migration, especially in osteosarcoma; WT1 was found to exert an antitumor effect in hematological and kidney diseases.^[28–32]^ Given their role in tumorigenesis, some researchers have investigated them in the differential diagnosis of SCST.^[33–35]^ As in granulosa cell tumors, we found value in their identification in pure stromal tumors, especially between LTSP and thecoma. We could assume that they are expressed more highly in thecoma cells resulting in lower invasion ability. However, the 4 other markers ESR, PGR, Vimentin, and HER2 had no value for discrimination, with a possible reason being that they are all associated with transcription and tumorigenesis in ovary tumors.

Our study had the advantage that results of IHC were compared quantitatively as AOD instead of commonly-used semiquantitative methods, maximally avoiding the biases of subjectivity. Furthermore, our research discovered and verified the MGAT5B-NCOA3 fusion gene at multiple levels to ensure the accuracy of the determination. Considering the limitations related to the rarity of the disease, further retrospective studies into the nature and absence of other markers such as calretinin, inhibin, and neural cell adhesion molecule 1 are warranted. More new cases and a wider range of indicators should be included in future studies.

The identified significant molecular pathological markers could help gynecologists discriminate between LTSP and thecoma quickly and accurately, so as to formulate targeted treatment strategies. In addition, the first discovery of the MGAT5B-NCOA3 fusion gene could strengthen the understanding of pathogenesis to treat or even prevent abdominal complications. When more fresh LTSP cases are collected, sequencing experiments should be performed to further reveal potential mechanisms.

## 5. Conclusions

In conclusion, our results shed further light on the pathological discrimination between LTSP and thecoma with significant molecular pathological markers containing MGAT5B, NCOA3, MKI67, β-Catenin, CD99, and WT1 by IHC, helping clinicians develop accurate treatment strategies. Moreover, we identified the MGAT5B-NCOA3 fusion gene for the first time, providing a new perspective on abdominal complications in LTSP patients.

The study was funded by the National Natural Science Foundation of China (NSFC) (81772775).

## Author contributions

**Conceptualization:** Juncheng Wei.

**Data curation:** Jia Liu.

**Formal analysis:** Jia Liu.

**Funding acquisition:** Juncheng Wei.

**Investigation:** Jia Wei.

**Methodology:** Jia Liu, Yiqun Yang.

**Project administration:** Jia Wei.

**Resources:** Juncheng Wei.

**Software:** Yiqun Yang.

**Supervision:** Juncheng Wei.

**Writing – original draft:** Jia Liu.

**Writing – review & editing:** Juncheng Wei.

## Supplementary Material




