# Supplementary material for: Pathological discrimination between luteinized thecoma associated with sclerosing peritonitis and thecoma
Source: Medicine (Baltimore). 2023 Jun 9;102(23):e33911. doi: 10.1097/MD.0000000000033911 (PMC10256399; doi:10.1097/MD.0000000000033911)

Supplemental Figure S1. Molecular pathological markers express no significant difference between LTSP and thecoma. **A-H**: immunohistochemical staining of estrogen receptor (ESR), progesterone receptor (PGR), Vimentin and receptor tyrosine-protein kinase erbB-2 (HER2) show equally intensity in the luteinized cells (**A**, **C**, **E**, **G**) and the thecoma cells (**B**, **D**, **F**, **H**). **I**: Statistical analysis of the four markers. AOD, average optical density; LTSP, luteinized thecoma associated with sclerosing peritonitis. Data are expressed as mean ± SD. n = 9–11 sections per marker (**I**, LTSP); n = 80–87 sections per marker (**I**, Thecoma). Scale bar, 500 μm.

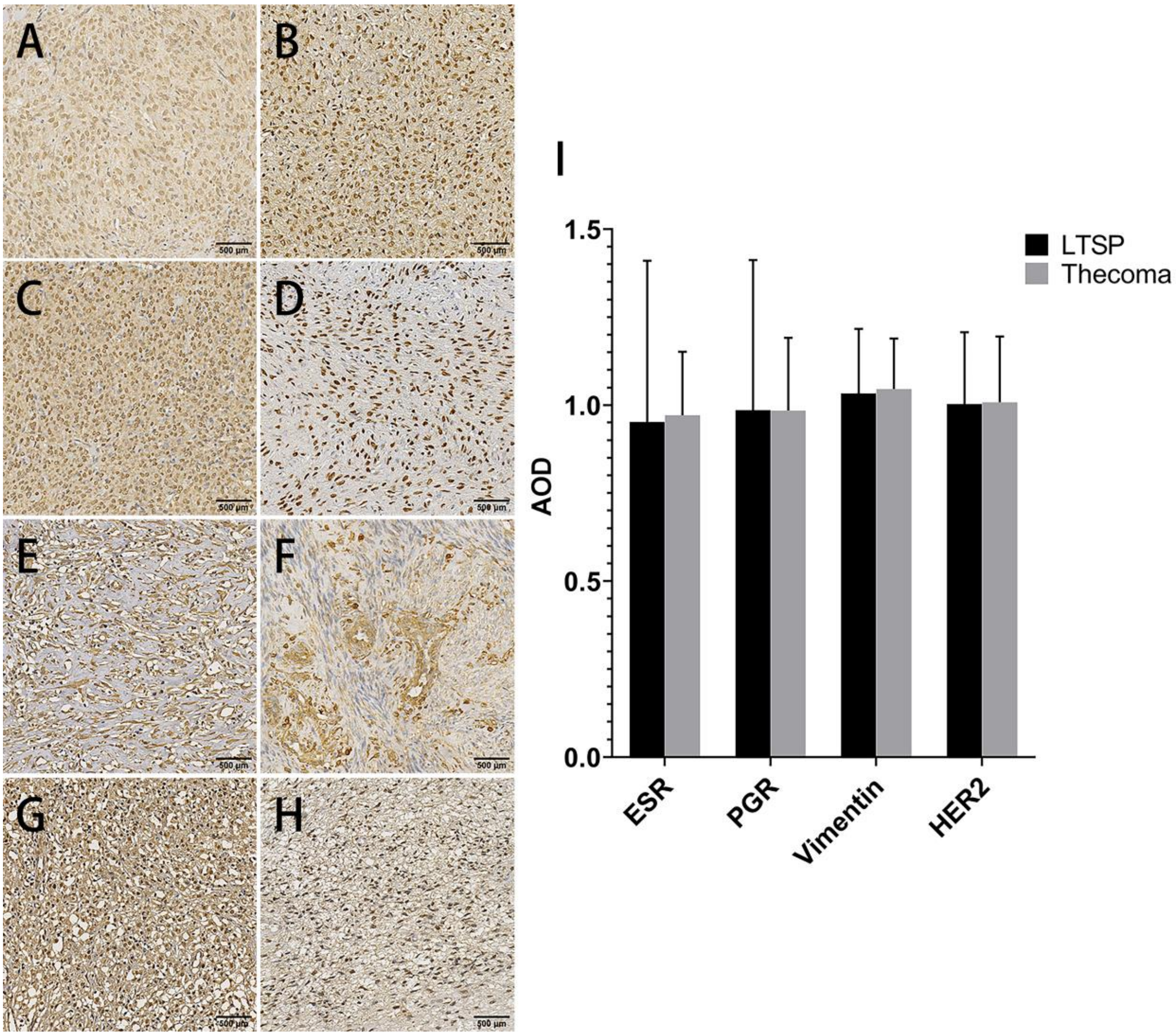

Supplement: Supplementary file 1 [file medi-102-e33911-s001.pdf]
