# Supplementary material for: Pathological discrimination between luteinized thecoma associated with sclerosing peritonitis and thecoma
Source: Medicine (Baltimore). 2023 Jun 9;102(23):e33911. doi: 10.1097/MD.0000000000033911 (PMC10256399; doi:10.1097/MD.0000000000033911)

Supplemental Figure S2. The sequences near breakpoints of MGAT5B and NCOA3 gene fragments are fused together.

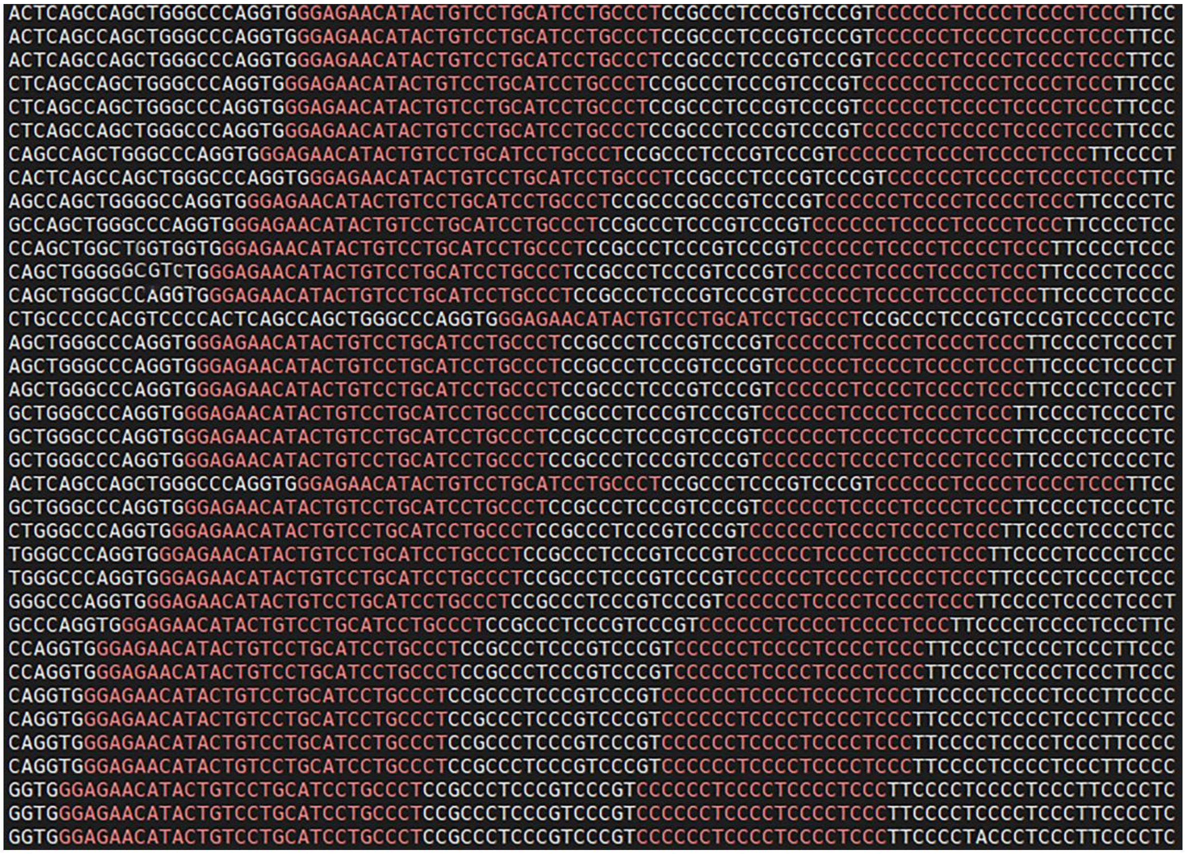

Supplement: Supplementary file 2 [file medi-102-e33911-s002.pdf]
